# CircPUM1 Knockdown Confers Radiosensitivity in Oral Squamous Cell Carcinoma by Regulating the miR-580/STAT3 Pathway

**DOI:** 10.3389/fgene.2022.907219

**Published:** 2022-08-25

**Authors:** Linghui Jia, Pengcheng Huang, Tingting Lin, Chunyan Lin, Xiaofen Ding, Liping Lin, Lifeng Zhu, Zhilian Zhou

**Affiliations:** ^1^ Fujian Key Laboratory of Oral Diseases, Fujian Provincial Engineering Research Center of Oral Biomaterial of Stomatological Key laboratory of Fujian College and University, School and Hospital of Stomatology, Fujian Medical University, Fuzhou, China; ^2^ Department of Prosthodontics, School and Hospital of Stomatology, Fujian Medical University, Fuzhou, China; ^3^ Department of Orthodontics, School and Hospital of Stomatology, Fujian Medical University, Fuzhou, China; ^4^ Affiliated Sanming First Hospital, Fujian Medical University, Sanming, China

**Keywords:** oral squamous cell carcinoma, radiosensitivity, CircPUM1, mir-580, STAT3

## Abstract

**Background:** CircPUM1 acts as an oncogene in a variety of tumors, and there is no related research on oral squamous cell carcinoma. This study aimed to evaluate the clinical significance of CircPUM1 in oral squamous cell carcinoma radiotherapy.

**Methods:** Radio-resistant cell lines were established by increasing the X-ray dose. Analysis of CircPUM1 expression in oral squamous cell carcinoma was carried out using bioinformatics tools. Cell proliferation was analyzed with CCK-8 and colony formation. Protein and gene expressions were detected by Western blotting and qPCR. RNA interference inhibits endogenous gene expression. A luciferase reporter system and immunoprecipitation were used to validate the target of CircPUM1.

**Result:** CircPUM1 was highly expressed in OSCC. The higher the expression level of CircPUM1 in OSCC, the worse the clinical features and prognosis. Knockdown of CircPUM1 enhances the sensitivity of OSCC cells to X-rays, and expression of exogenous CircPUM1 makes OSCC cells acquire radiation resistance. The absence of CircPUM1 blocked the cells in the G0/G1 phase and triggered apoptosis. The prediction of mir-580-binding site, luciferase reporter system, and immunoprecipitation confirmed that mir-580 is the binding site of CircPUM1. In addition, STAT3 was predicted and confirmed as the binding site of mir-580. Overexpression of STAT3 partially attenuated the radiosensitivity of OSCC cells to knockdown of CircPUM1.

**Conclusion:** CircPUM1 has the oncogene expression profile in oral squamous cell carcinoma; patients with high expression of CircPUM1 have less benefit from radiotherapy and need more frequent follow-up. In addition, CircPUM1 may be a potential therapeutic target for oral squamous cell carcinoma. The CircPUM1/mir-580/STAT3 axis has a certain effect on the radiosensitivity of OSCC. These results suggest that patients with low expression of CircPUM1 may gain more benefits.

## Introduction

Oral squamous cell carcinoma is one of the most common malignant tumors in oral and maxillofacial regions (Müller, 2017; Kennedy, 2018). Oral squamous cell carcinoma has poor clinical prognosis and low survival rate (Al-Sarraf, 2007). Currently, the combined treatment of surgery plus radiotherapy and chemotherapy can significantly improve the overall survival rate of patients with oral squamous cell carcinoma (Aslam et al., 2012). Radiotherapy, one of the curative treatments for oral squamous cell carcinoma, can be used with surgery, chemotherapy, or alone (Pentenero et al., 2005). Radiotherapy, an important part of comprehensive sequential therapy for advanced oral squamous cell carcinoma, can improve the local control rate (Berdugo et al., 2019). However, improving the effect of radiotherapy for oral squamous cell carcinoma is the main problem that we are facing currently.

Studies have shown that circular RNA (circRNA) is a kind of non-coding RNA, which has high stability due to its closed circular molecule (Zheng et al., 2019). Studies have shown that circRNA can inhibit its expression by acting as a sponge molecule of microRNA (miRNA), thus regulating the biological processes of tumor cell proliferation, apoptosis, migration, and so on (Lee et al., 2021). However, there are relatively few studies on circRNAs in oral squamous cell carcinoma. Circular RNA pum1 (circular RNA pum1) is highly expressed in lung cancer, ovarian cancer, colon cancer, and other tumors and can promote the occurrence and development of tumors (Jeck et al., 2013; Salzman et al., 2013; Rybak-Wolf et al., 2015). CircPUM1 (circ_0000043) is highly expressed in endometrial adenocarcinoma (Deng et al., 2020), ovarian adenocarcinoma (Guan et al., 2019), and lung adenocarcinoma (Chen et al., 2019) and plays a role as an oncogene, but it has not been reported in oral squamous cell carcinoma. Circinteractome predicted that CircPUM1 targeted mir-580 (the highest predicted score was 99). starBase predicted that mir-580 targeted Stat3. STAT3 has been reported to be involved in radiosensitivity of oral squamous cell carcinoma (Yu et al., 2020).

However, it is not known whether CircPUM1 affects the malignant biological behavior of oral squamous cells by targeting the expression of mir-580/Stat3. Therefore, this study mainly explored the expression of CircPUM1 and mir-580/STAT3 in oral squamous cell carcinoma and analyzed whether CircPUM1 regulates radiosensitivity through mir-580/STAT3. Therefore, we believe that our findings will provide a theoretical basis for improving the radiosensitivity of OSCC.

## Materials and Methods

### Patients and Sample

A total of 50 OSCC and matched normal tissues were collected. All samples were independently confirmed by two pathologists. Immediately after operation, the specimens were frozen in liquid nitrogen and then stored in the refrigerator at − 80 C for future use. Patients who received any neoadjuvant therapy and had a history of cancer were excluded from the study. Prior to the start of the study, informed consent was obtained from the participants, and the study was conducted in strict accordance with the Helsinki declaration.

### Cell Culture

Human oral squamous cell carcinoma cell lines Cal-27 and hsc3 were cultured in DMEM medium containing 10% fetal bovine serum, 100 units/ml penicillin–streptomycin, and 100% glucose at 37°C and 5% CO_2_.

### Irradiation of Cells and Development of Radio-Resistant Cell Lines

The cells were irradiated using the Faxitron cabinet X-ray system 43855d (Faxitron X-ray Company, IL, USA). Mcf-7r and mda-mb-231r were established on their parent cell lines. In short, the initial dose was 2 Gy, then the dose increased by 0.5 per week, and the total radiation dose reached 57 Gy in 12 weeks. After that, 5 Gy per week was used for further maintenance.

### Cell Transfection

The shRNA was synthesized and subcloned into a plko.1-trc-puro plasmid. The sequence of shRNA is shown in Table 1. We used the Lipofectamine 2000 system to transfect the plasmid. In short, 8 × 105 cells were seeded in a six-well dish for 24 h, and then Liposome 2000 was mixed with 5 μg plasmid. Using Opti-MEM, the mixture was successively incubated at room temperature for 30 min, then the mixture was added to each well, supplemented with Opti-MEM to 1 ml, and incubated for 6 h. After 6 h, DMEM containing 20% FBS was added to each well and 2 ml medium and then cultured for 48 h for mRNA extraction and protein collection. According to the results predicted by Circinteractome, in order to overexpress CircPUM1, the whole coding sequence 5 ‘GAC​UUU​UUG​ACU​ACA​AUU​CUC​AA3′ of CircPUM1 was subcloned into the pZsG vector.

**TABLE 1:** Patient information and clinicopathological characteristics of 58 patients with OSCC *P < 0.05 or **P < 0.01 was considered significant (chi‐square test between 2 groups).

Patients	Low circPUM1 (*n* = 29)	High circPUM1 (*n* = 29)	*P*-value
Age(years)			0.11556
≥65	16	17	
<65	13	12	
Gender			0.06496
Female	14	17	
Male	15	12	
Remote metastasis			0.00049**
No	9	21	
Yes	20	8	
Lymph node metastasis			0.00025**
No	7	19	
Yes	22	10	
TNM satge			<0.01**
I	3	6	
II	5	11	
III	8	8	
IV	13	4	
Differentiation			0.07813
Poor	12	11	
Moderate	5	4	
Well	13	14	

### RNA Extraction and Quantitative Real-Time Polymerase Chain Reaction (QRT-PCR) Analysis

According to the manufacturer’s protocol, TRIzol reagent (Invitrogen, Carlsbad, CA) was used to extract total RNA. The first-strand cDNA was synthesized by a reverse transcription kit (Takara, Dalian, China). GAPDH is used as an internal control. PCR primers for CircPUM1 and 18S RNA were found in starBase prediction. The relative expression of CircPUM1 was expressed with the 2^-ΔΔCT^ method. All samples are in triplicate.

### Cell Proliferation Assay

Cell proliferation test: Cal-27 and HSC3 cells were seeded onto a 96-well plate (Müller, 2017) and cultured for 24, 48, and 72 h. The incubation time was 37 h. μ CCK8 was injected into each well and incubated at 37°C. The absorbance at 480 nm was measured using the Rayto-6000 system (Rayto, China) after 2 h of storage of C and normalized to DMEM medium as control.

### Flow Cytometry Analysis

In cell cycle analysis, the cells were harvested after 6 h of starvation, fixed overnight with cold ethanol, and then incubated in the dark with propidium iodide and ribonuclease (BD, USA) for 15 min. In apoptosis analysis, after 6 h of starvation, the cells were washed twice with cold PBS, stained with FITC-binding Annexin V for 20 min, and stained with propidium iodide for 15 min. The stained cells were detected by flow cytometry (FACSAria III, BD, USA) and analyzed by FlowJo vx 0.7 software.

### Western Blot Analysis

Protein was extracted with RIPA lysis buffer containing protease inhibitor (Roche). Quantitative analysis of protein was conducted using BCA ™ protein analysis kit (Pierce, Appleton, Wisconsin, USA). After that, protein (30 μg/sample) was transferred to a poly (vinylidene fluoride two fluoroethylene) membrane by 10% alkyl sulfate-polyacrylamide gel electrophoresis.

The antibody was prepared in 5% blocking buffer with a dilution of 1:1,000, incubated with the membrane at 4 °C overnight, washed twice with TBST, then cultured with a secondary antibody (1:2000), and labeled with horseradish peroxidase for 2 h at room temperature. Immuno Western chemiluminescence HRP substrate (Millipore) was used to cover the film surface. Finally, the signal was captured, and the concentration of the band was quantified by Image Lab ™ software (Bio-Rad Laboratories, Hercules, CA, USA).

### Luciferase Report Analysis

Methods: circPUM1-WT and circPUM1-Mut were subcloned into pGL3 vectors (Promega, Madison, WI) to construct plasmids. The plasmids were further transfected with designated mimics or siRNA for 48 h. The PCR products were cloned into the polyclonal sites of the recombinant pGL3 expression vector. Finally, the luciferase activity was detected by Dual Luciferase Report Analysis kit (Promega, Madison, Wisconsin, USA).

### Statistical Analysis

All statistical analyses were performed using GraphPad Prism version 8.0 (GraphPad Software, La Jolla, California). The significant differences between groups were estimated using Student’s t-test. A *p*-value less than 0.05 was considered statistically significant. The results are reported as average ± standard deviation. All experiments were carried out in triplicate.

## Results

### CircPUM1 Was Highly Expressed in OSCC

**FIGURE 1:**
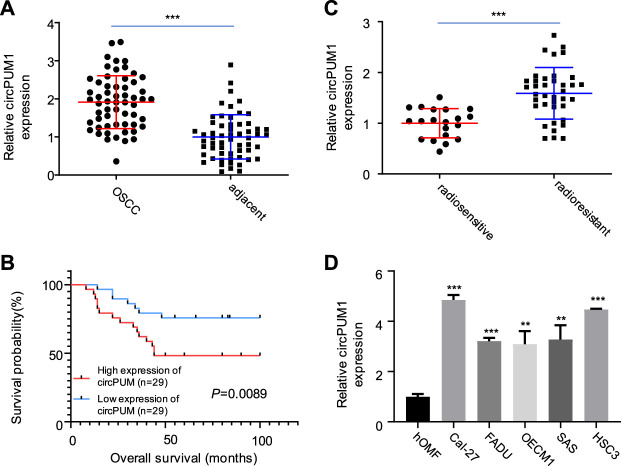
Expression of CircPUM1 in OSCC carcinoma and adjacent tissues **(A)**; overall survival rate of low-expression group and high-expression group of CircPUM1 **(B)**; expression of CircPUM1 in OSCC tissues with different radiation doses **(C)**; expression of CircPUM1 in different OSCC cells **(D)**. *p* < 0.01 (∗∗) and *p* < 0.001 (∗∗∗).

First, we analyzed the expression of CircPUM1 in patients. QRT-PCR was used to detect the expression level of CircPUM1 in 58 pairs of OSCC cancer tissues and corresponding adjacent tissues. It was found that the expression level of CircPUM1 in OSCC was significantly increased (*p* < 0.001, Figure 1A). At the same time, 58 OSCC patients were divided into two groups: low-expression group (n = 29) and high-expression group (n = 29) according to the cut-off value of median expression of CircPUM1 in Figure 1A. Chi square test was used to analyze the relationship between the expression of circpum1 and the clinicopathological data of OSCC, *p* < 0.05, but not related to the patient's age, gender and tumor differentiation. The Kaplan–Meier survival curve was used to evaluate the overall survival rate of the two groups. It was found that the prognosis of high-expression group of CircPUM1 was poor, and the difference was statistically significant (*p* = 0.0089, Figure 1B). Subsequently, the expression levels of CircPUM1 in radiosensitive (n = 20) and radio-resistant (n = 38) were detected by QRT-PCR. The expression of CircPUM1 in radio-resistant OSCC was significantly increased (*p* < 0.001, Figure 1C). As mentioned previously, we successfully established the expression level of CircPUM1 in two radiation-resistant OSCC cell lines (Cal-27, FaDu, OECM1, SAS, and HSC3) and the human oral mucous fibroblasts (HMFs). The expression level of CircPUM1 in OSCC cell lines was higher than that in normal tissues (*p* < 0.001, Figure 1D).

### Knockdown of CircPUM1 Enhances Radiosensitivity of OSCC Cells

**FIGURE 2:**
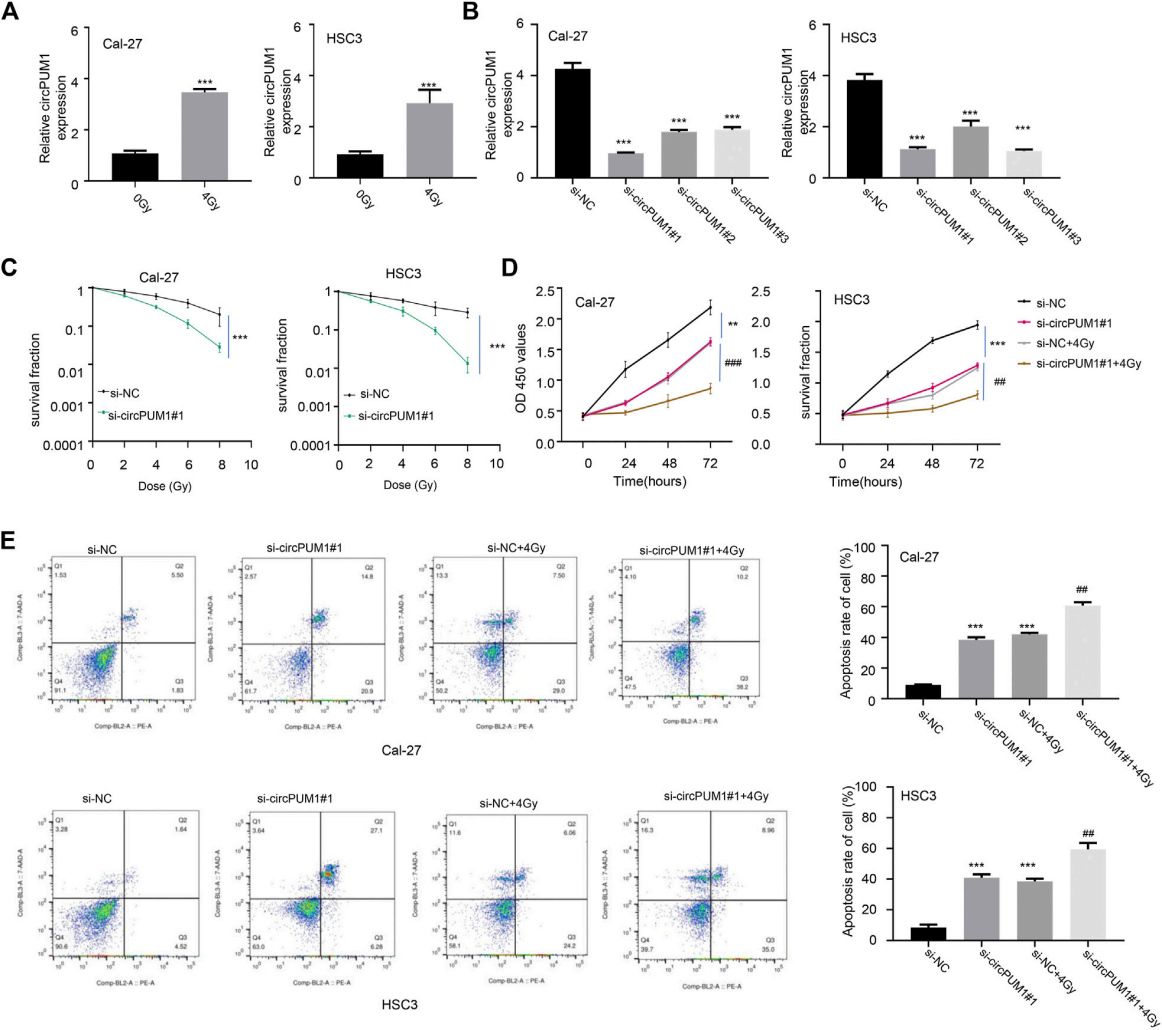
QRT-PCR was used to detect the expression of CircPUM1 in Cal-27 and hsc3 cells**(A)**; expression of CircPUM1 in different groups of OSCC cells **(B)**; colony survival fraction of Cal-27 and hsc3 cells in different groups under different radiation doses**(C)**; optical absorption values of Cal-27 and hsc3 cells in different groups at 450 nm at different time periods were measured**(D)**; apoptosis was observed in different groups of Cal-27 and hsc3 cells**(E)**. *p* < 0.01 (∗∗) and *p* < 0.001 (∗∗∗).

We transfected the CircPUM1 siRNA and expression plasmid into Cal-27 and hsc3 cells (*p* < 0.005, Figures 2A,B). The cells were exposed to different doses of ionizing radiation (0 and 4 Gy), and the expression level of CircPUM1 increased after 4 Gy; the difference was statistically significant. QRT-PCR was used to detect the expression level of CircPUM1 in Cal-27 and hsc3 cells of different groups (Si-nc, si-CircPUM1 # 1, si-CircPUM1 # 2, and si-CircPUM1 # 3). Colony formation assay was used to detect the colony survival fraction of Cal-27 and hsc3 cells in different groups (Si-nc and si-CircPUM1 # 1) under different radiation doses (0, 2, 4, and 8 Gy). Compared with the Si NC group, the colony survival fraction of si-CircPUM1 # 1 cells gradually decreased with the gradual increase in radiation dose Gy (*p* < 0.005, Figure 2C). The results confirmed that radiosensitivity was a dose-dependent inhibitory effect. The loss of function of CircPUM1 induced more apoptosis when exposed to 4 Gy X-ray.

CCK8 was used to detect the light absorption values of Cal-27 and hsc3 cells in different groups (Si-nc, si-CircPUM1#1, Si-nc + 4Gy, and si-CircPUM1#1 + 4Gy) at the wavelength of 450 nm at 0h, 24h, 48h, and 72 h. Compared with the Si NC group, the light absorption values of si-CircPUM1#1 and Si-nc + 4Gy groups were lower at the wavelength of 450nm; compared with the Si NC + 4Gy group, the optical absorption value of si-CircPUM1 # 1 + 4Gy at 450 nm wavelength decreased, and the difference was statistically significant (*p* < 0.01, Figure 2D). The apoptosis level of Cal-27 and hsc3 cells in different groups (Si NC, si-CircPUM1 # 1, Si NC + 4Gy, and si-CircPUM1 # 1 + 4Gy) was detected by flow cytometry. Compared with the Si NC group, the apoptosis level of si-CircPUM1 # 1 and Si-nc + 4Gy groups was high; compared with the Si NC + 4Gy group, the apoptosis level of si-CircPUM1 # 1 + 4Gy group was significantly high (*p* < 0.01, FIG.2E). The results showed that the low expression of CircPUM1 could promote the radiosensitivity and apoptosis of OSCC tumor cells.

### CircPUM1 Targets mir-580 and Inhibits Its Expression in OSCC Cells

**FIGURE 3:**
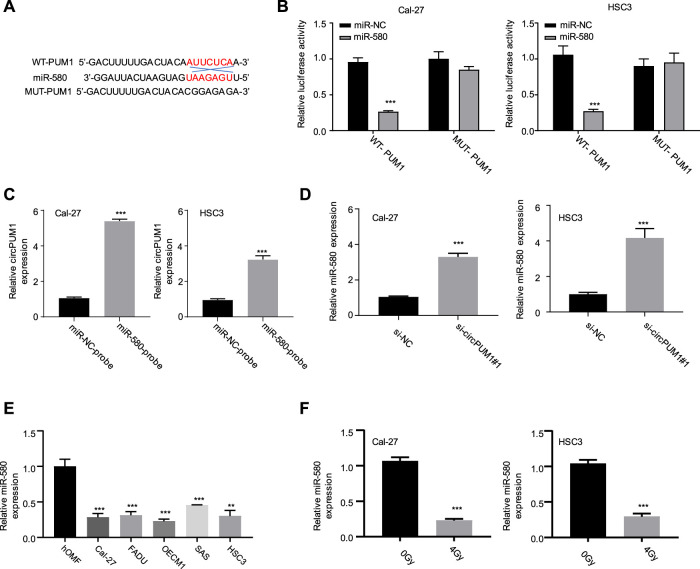
Binding site of CircPUM1 and mir-580 **(A)**; effect of mir-580 on the activity of CircPUM1 **(B,C)**; relationship between knockdown of CircPUM1 and mir-580 expression **(D)**; expression level of mir-580 in the OSCC cell line **(E)**; expression of mir-580 in OSCC cells after 4 Gy radiation **(F)**. *p* < 0.01 (∗∗) and *p* < 0.001 (∗∗∗).

Next, we want to know how CircPUM1 plays its biological function in radiation-resistant cells. Through the analysis of the Circinteractome online database, we found that there was a mir-580-binding site in CircPUM1. Luciferase reporter gene experiments were carried out in Cal-27 and hsc3 cells, respectively. The results showed that overexpression of mir-580 could inhibit the luciferase activity of wild-type CircPUM1 vector in Cal-27 and hsc3 cells, compared with mir-nc. After mutating the predicted mir-580-binding site, the inhibitory effect disappeared (Figures 3A,B); compared with the NC probe, the mir-580 probe enriched more CircPUM1 in Cal-27 and hsc3 cells (Figure 3C). QRT-PCR was used to detect the expression level of mir-580 in Cal-27 and hsc3 cells in different groups (Si-nc, si-CircPUM1 # 1). Knockdown of CircPUM1 increased the level of mir-580 in cells, and the difference was statistically significant (*p* < 0.01, Figure 3D). The results showed that the activity of CircPUM1 was negatively correlated with the expression of mir-580. QRT-PCR was used to detect the expression of mir-580 in OSCC cell lines (Cal-27, FaDu, OECM1, SAS, and HSC3) and the human oral mucous fibroblasts (HMFs). The expression of mir-580 in OSCC cell lines was low, and the difference was statistically significant (*p* < 0.01, Figure 3E). QRT-PCR was used to detect the expression level of mir-580 in Cal-27 and hsc3 cells in different groups (0gy, 4Gy). After 4Gy radiation, the expression level of mir-580 decreased, and the difference was statistically significant (*p* < 0.001,Figure 3F).

### STAT3 Is a Downstream Target Gene of miR-580

**FIGURE 4:**
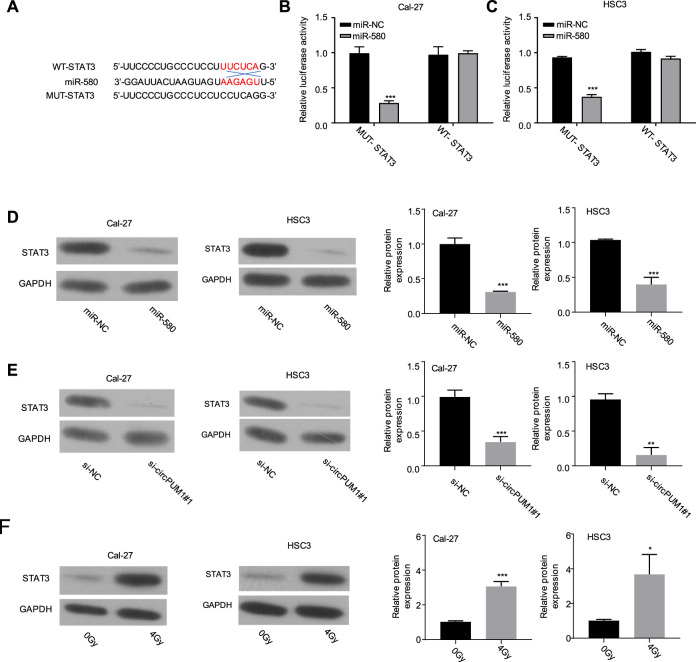
Binding site of mir-580 and STAT3 **(A)**; luciferase reporter gene assay verified the targeting relationship between mir-580 and STAT3 **(B,C)**; protein expression level of STAT3 after mir-580 overexpression **(D)**; knockdown of CircPUM1 and STAT3 protein expression in the OSCC cell line **(E)**; expression of STAT3 in OSCC cells after 4 Gy irradiation **(F)**. *p* < 0.05 (∗), *p* < 0.01 (∗∗), and *p* < 0.001 (∗∗∗).

After that, we analyze the target of CircPUM1 through starBase. Among the targets found, STAT3 scored higher, which predicted that mir-580 and STAT3 had binding sites, as shown in Figure 4A. Luciferase reporter gene experiments were carried out in Cal-27 and hsc3 cells to verify the targeting relationship. Compared with mir-nc, overexpression of mir-580 could inhibit the activity of luciferase in cells, and the inhibition disappeared after mutating the predicted STAT3-binding site (*p* < 0.001, Figures 4B,C). Our samples showed that the mir-580 expression level was negatively correlated with STAT3 expression. WB detected the protein expression level of STAT3 after overexpression of mir-580 in Cal-27 and hsc3 cells. Compared with mir-nc, mir-580 overexpression downregulated the protein expression of STAT3, and the difference was statistically significant (*p* < 0.001, Figure 4D). WB was used to detect the protein expression level of STAT3 in Cal-27 and hsc3 cells of different groups (Si NC and si-CircPUM1 # 1). Knockdown of CircPUM1 downregulated the protein expression of STAT3, and the difference was statistically significant (*p* < 0.01, Figure 4E). WB method was used to detect the expression level of STAT3 in Cal-27 and hsc3 cells after different doses of ionizing radiation (0gy and 4Gy groups). The expression level of STAT3 increased after 4Gy radiation, and the difference was statistically significant ( *p* < 0.05,Figure 4F).

### Overexpression of STAT3 Can Partially Reverse the Effect of Knockdown of CircPUM1 on Radiosensitivity of OSCC Cells

**FIGURE 5:**
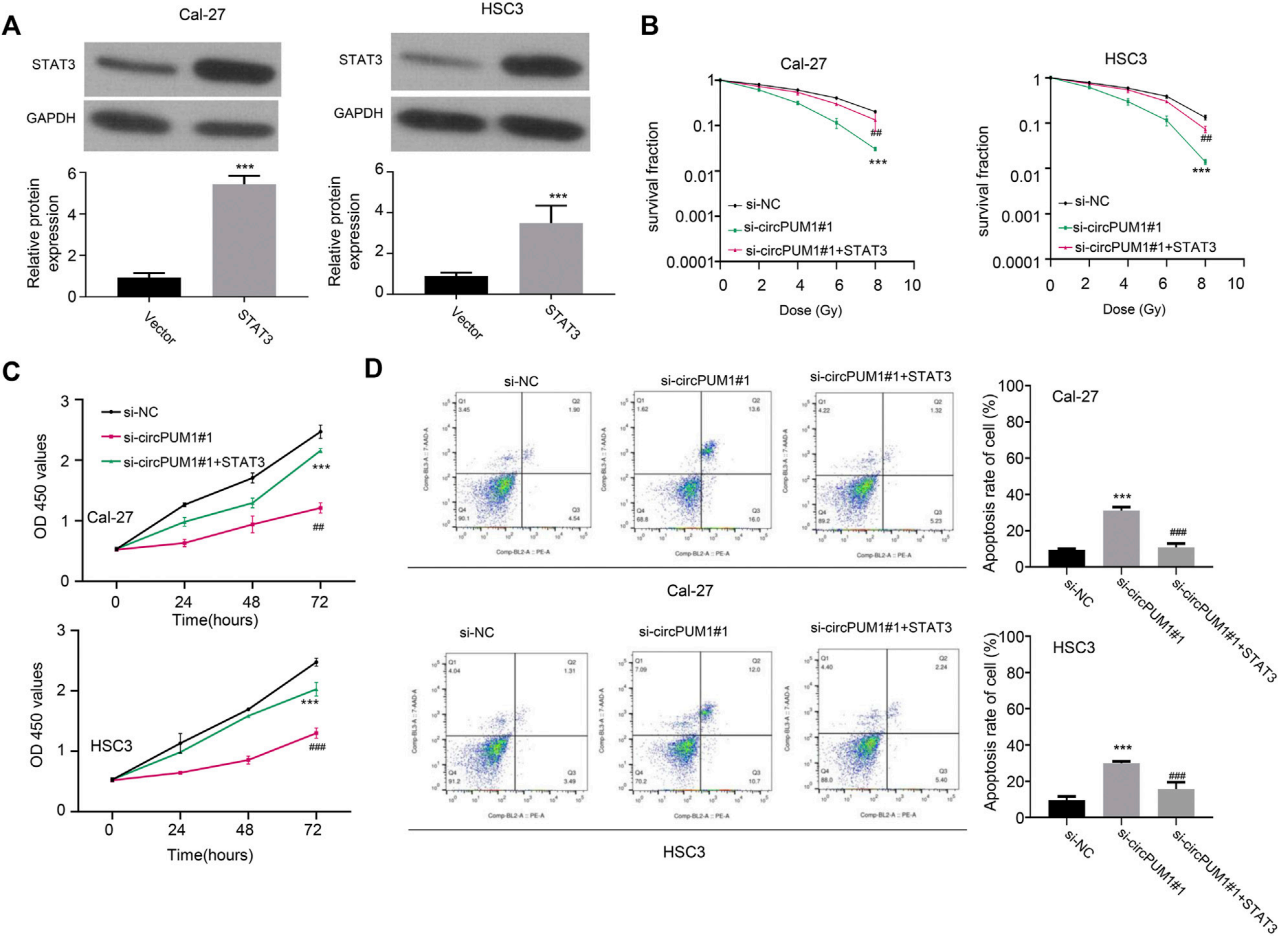
Overexpression of STAT3 protein in OSCC cells **(A)**; grouping of Cal-27 and hsc3 cells at 4Gy: the optical absorption values of Si-nc, si-CircPUM1#1, and si-CircPUM1#1 + STAT3 at 0h, 24h, 48h, and 72 h **(B,C)** at 450 nm wavelength of different groups of cells under different time periods of radiation; knockdown of si-CircPUM1#1 and cotransfection of STAT3 plasmid induced apoptosis **(D)**. *p* < 0.01 (∗∗) and *p* < 0.001 (∗∗∗).

The target of CircPUM1 is analyzed continuously by starBase. The predicted binding sites of CircPUM1 and STAT3 are shown in Figure 5A, *p* < 0.001. In addition, studies have shown that upregulation of STAT3 can promote radiotherapy tolerance. Therefore, this study investigated whether STAT3 affects the stability of CircPUM1 protein by regulating the expression of STAT3 in OSCC cells. First, we found that STAT3 was elevated in OSCC tissues and radiation-resistant cells (*p* < 0.001, Figures 5B,C). As mentioned previously, CircPUM1 deficiency can make cells sensitive to radiation. Knockdown of si-CircPUM1 # 1 increased the level of apoptosis. After cotransfection with the STAT3 plasmid, the level of apoptosis was partially decreased (*p* < 0.001, Figure 5D). Therefore, high expression of STAT3 could partially inhibit the effect of knockdown of CircPUM1 on radiosensitivity of OSCC cells.

## Discussion

Recent studies have shown that lncRNA plays a key regulatory role in the pathogenesis, progression, and phenotype development of oral squamous cell carcinoma (Xu et al., 2020). Accumulating data suggest that lncRNA plays a role in oral squamous cell carcinoma (Liu et al., 2017). As mentioned previously, CircPUM1 plays a role in the treatment of various cancers. In our review of published articles, CircPUM1 may be closely associated with the prognosis of colon cancer (Ju et al., 2019), ovarian cancer (Zhang et al., 2019), and gastric cancer (Li et al., 2019a). Scholars have expounded on the different mechanisms of CircPUM1 in different cancers, including regulating the mir-524-5p axis of colon cancer, circc3p1/mir-21, tumor-suppressor gene *PTEN*, and NF-κB and PI3K/Akt pathways (Zhao et al., 2016; Su et al., 2020). However, the role of CircPUM1 in oral squamous cell carcinoma is rare.

CircRNAs are a group of non-coding RNAs with stable closed-loop structures that prevent them from being broken down by enzymes (Li et al., 2019b). Accumulating data suggest that circRNAs are closely related to the progression of NSCLC (Yu et al., 2019). For example, inhibition of circRNA VANGL1 can inhibit bladder cancer progression (Yang et al., 2020). Circ_000984 promotes cell proliferation and metastasis in NSCLC by regulating the Wnt/β-catenin pathway (Li et al., 2019c). Some researchers found that (Pang et al., 2020) the expression of circ_0072309 was downregulated in NSCLC tissues and cells, and the overexpression of circ_0072309 significantly prevented the proliferation, migration, and invasion of cells, which indicated that circ_0072309 played a tumor-suppressor role in NSCLC.

The circular PUM1 RNA (circPUM1, has_circ_0000043) is derived from exon backsplicing of the *PUM1* gene. Recent studies have shown that circPUM1 is highly expressed in endometrial cancer, lung adenocarcinoma, and ovarian cancer tissues (Chen et al., 2019; Guan et al., 2019; Zong et al., 2020). Furthermore, circPUM1 can inhibit tumor development through cavernous microRNAs. These studies suggest that circPUM1 may play an important role in the development of diseases such as tumors. Related studies have reported that circPUM1 can promote the proliferation, invasion, and migration of HCC *in vitro*, and studies have shown that circPUM1 can act as an oncogene of HCC (Zhang et al., 2021). In conclusion, circPUM1 may function as an oncogene in human cancers. In our data, we found that reduction of CircPUM1 induced apoptosis of OSCC cells and enhanced their radiosensitivity.

Aberrant expression of miR-580 in many tumors, such as glioma and breast cancer, has been investigated (Loberg et al., 2006). Also, studies have shown that the level of miR-580 in tumor tissue is significantly higher than that in adjacent normal tissue, which can promote the proliferation, invasion, and migration of HCC (Wang et al., 2021). Mir-580 was identified as a potential target of CircPUM1. Among the discovered targets, mir-580 and CircPUM1 had higher binding sites and predicted values. Furthermore, our data suggest that CircPUM1 can competitively adsorb mir-580 to enhance STAT3 expression in OSCC cells. After we blocked the function of STAT3, OSCC cells triggered the apoptotic pathway and restored their radiosensitivity. This phenotype can be obtained by exogenous mir-580 expression or STAT3 deletion.

Many studies have shown that constitutive STAT3 is activated in a variety of human tumors (Grandis et al., 2000). Evidence suggests that abnormal STAT3 signaling promotes the occurrence and development of human cancers by inhibiting apoptosis, inducing cell proliferation, angiogenesis, invasion, and metastasis (Melinda et al., 2000; Leong et al., 2003; Bollrath et al., 2009; Zhu et al., 2019; Chen et al., 2020), as well as inducing inflammation and immunosuppression (Leaman et al., 1996; Mohan et al., 2022; Tse et al., 2022). In OSCC studies, STAT3 is the most common signal transducer and activator of transcription. STAT3 plays a variety of biological effects in the degree of invasion, lymph node metastasis, and different clinical grades of oral squamous cell carcinoma. It is of great value for early diagnosis and can be used as an important biological indicator for judging prognosis.

## Data Availability

The original contributions presented in the study are included in the article/Supplementary Material; further inquiries can be directed to the corresponding authors.
